# The effect of education and supervised exercise on physical activity, pain, quality of life and self-efficacy - an intervention study with a reference group

**DOI:** 10.1186/s12891-018-2098-3

**Published:** 2018-06-21

**Authors:** Thérése Jönsson, Eva Ekvall Hansson, Carina A. Thorstensson, Frida Eek, Patrick Bergman, Leif E. Dahlberg

**Affiliations:** 1 Department of Clinical Sciences Lund, Orthopedics, Skane University Hospital, Lund University, Lund, Sweden; 2 BOA Registry, Centre of Registries, Västra Götaland, Gothenburg, Sweden; 3 Department of Health Sciences, Division of Physiotherapy, Lund University, Lund, Sweden; 4 Department of Clinical Neuroscience and Rehabilitation, Institute of Neuroscience and Physiology, Sahlgrenska Academy, University of Gothenburg, Gothenburg, Sweden; 5 Department of Sport Science, Linnaeus University, Kalmar, Sweden

**Keywords:** Osteoarthritis, Knee, Hip, Patient education, Exercise, Physical activity, Accelerometer

## Abstract

**Background:**

Individuals with knee and hip osteoarthritis (OA) are less physically active than people in general, and many of these individuals have adopted a sedentary lifestyle. In this study we evaluate the outcome of education and supervised exercise on the level of physical activity in individuals with knee or hip OA. We also evaluate the effect on pain, quality of life and self-efficacy.

**Methods:**

Of the 264 included individuals with knee or hip OA, 195 were allocated to the intervention group. The intervention group received education and supervised exercise that comprised information delivered by a physiotherapist and individually adapted exercises. The reference group consisted of 69 individuals with knee or hip OA awaiting joint replacement and receiving standard care. The primary outcome was physical activity (as measured with an accelerometer). The secondary outcomes were pain (Visual Analog Scale), quality of life (EQ-5D), and self-efficacy (Arthritis Self-Efficacy Scale, pain and other symptoms subscales). Participants in both groups were evaluated at baseline and after 3 months. The intervention group was also evaluated after 12 months.

**Results:**

No differences were found in the number of minutes spent in sedentary or in physical activity between the intervention and reference groups when comparing the baseline and 3 month follow-up. However, there was a significant difference in mean change (mean diff; 95% CI; significance) between the intervention group and reference group favoring the intervention group with regard to pain (13; 7 to 19; *p* < 0.001), quality of life (− 0.17; − 0.24 to − 0.10; *p* < 0.001), self-efficacy/other symptoms (− 5; − 10 to − 0.3; *p* < 0.04), and self-efficacy/pain (− 7; − 13 to − 2; *p* < 0.01). Improvements in pain and quality of life in the intervention group persisted at the 12-month follow-up.

**Conclusions:**

Participation in an education and exercise program following the Swedish BOA program neither decreased the average amount of sedentary time nor increased the level of physical activity. However, participation in such a program resulted in decreased pain, increased quality of life, and increased self-efficacy.

**Trial registration:**

The trial is registered with ClinicalTrials.gov. Registration number: NCT02022566. Retrospectively registered 12/18/2013.

## Background

Osteoarthritis (OA) in the knee and hip is estimated as the eleventh highest contributor to global disability [1]. Increasing life expectancy and increasing prevalence of obesity and sedentary lifestyles (two known risk factors for OA) suggest that the number of people living with hip or knee OA will grow substantially over the coming decades [2]. Pain, stiffness, and functional impairments are common complaints, resulting in limitations to activity and decreased quality of life [3]. In addition, people with OA are less physically active (PA) and more sedentary than the general population [4], leading to an increased risk of cardiovascular diseases and premature death [5].

The primary goals of OA management are to reduce pain, improve functional ability and quality of life, and increase PA level. According to OA guidelines, patient education and individualized exercise are core treatments [6–8]. Total joint replacement should only be considered when nonsurgical treatments have been tried and failed. Despite the fact that evidence-based guidelines have existed for some 10 years [9], statistics from 2015 demonstrate that only 70% of patients receiving a total hip replacement in Sweden have been treated by a physiotherapist (PT), whereas only 34% had access to standardized information and exercise through a self-management program for OA [10]. We can divide barriers to adhering to OA treatment guidelines into four themes: “OA is not that serious,” reflecting an attitude that everybody eventually gets OA as they grow older, “Clinicians are, or perceive they are, under-prepared,” “Personal beliefs at odds with providing recommended practice,”, and “Dissonant patient expectations” [11]. Factors that facilitate improved adherence to the guidelines are patient-tailored strategies to improve patients’ knowledge, self-management, and communication with healthcare professionals on matters such as shared decision making [12]. To overcome the discrepancy between the guidelines and practice, a nationwide program titled “Better management of patients with osteoarthritis” (BOA) was initiated in Sweden in 2008 to offer information and individually adapted exercise to all patients with hip and knee OA, in accordance to guidelines for OA [13]. The BOA program has three central components: patient education, training of healthcare professionals, and depositing patient-reported outcomes before and after treatment at the National Quality Register, the BOA registry.

Participants in OA self-management programs, including education both with and without exercise, have shown positive effects on patients’ reported outcome measures, such as pain [14, 15], quality of life [16–18], self-efficacy [14] and patient-reported PA [19]. However, it is still unclear if self-management programs for OA have an effect on objectively measured sedentary time or level of PA.

In individuals with knee OA being obese/overweight, quality of diet, severe knee pain, and knee dysfunction are factors associated with physical inactivity [20]. For this population, barriers to PA include pain, physical limitations, absence of positive experiences and beliefs of PA, a resigned attitude and distress due to OA, lack of behavioral regulation, lack of support from healthcare professionals, and negative social comparisons when exercising in a group [21]. Factors that have been shown to facilitate physical activity are symptom relief, increased mobility, positive exercise experiences, and beliefs, knowledge, enjoyment of exercise, a “keep going” attitude, adjusting to and prioritizing PA, and having professional and social support [21].

We hypothesize that individuals with knee or hip OA treated with education and supervised exercise according to the BOA program will decrease time in sedentary and increase time in different levels of physical activity. We also hypothesize that these participants will experience decreased pain, increased health related quality of life and increased self-efficacy following intervention, compared to a reference group receiving standard care.

The primary aim of this study was to evaluate the outcome of education and supervised exercise following the BOA program on the level of PA, measured by an accelerometer, in individuals with knee and hip OA. The secondary aims were to evaluate the effect of this program on pain, quality of life, and self-efficacy.

## Method

This study was conducted as an intervention study with a reference group. No randomization was performed, and the participants were consecutively recruited. The PT was not blinded with respect to the intervention group or reference group. The intervention group participated in education and supervised exercise following the BOA program; described in detail elsewhere [13]. The reference group receive standard care (i.e. not education and supervised exercise according to BOA) and were told not to make any lifestyle changes. At the 3 month follow-up, the reference group was offered education and supervised exercise. Individuals with knee or hip OA were included. The intervention group was recruited from the Departments of Orthopedics at Sahlgrenska University Hospital and Skåne University Hospital between 2009 and 2011. The reference group was recruited from the waiting list to see an orthopedic surgeon due to knee or hip pain at the Department of Orthopedics, Skåne University Hospital, between 2011 and 2014. Diagnosis of OA was confirmed by medical history and a physical examination based on the clinical criteria of the American College of Rheumatology [22, 23]. The inclusion criteria were clinically diagnosed OA and age between 18 and 75 years. Exclusion criteria were confirmed or suspected cancer, rheumatoid arthritis, hip fracture sequel, chronic pain or fibromyalgia, total joint replacement within the past 12 months, other surgery of the knee or hip joint within the past 3 months, and not being able to read or understand Swedish. All patients received oral and written information about the study and provided their written informed consent before inclusion.

### Education and supervised exercise

**Fig. 1:**
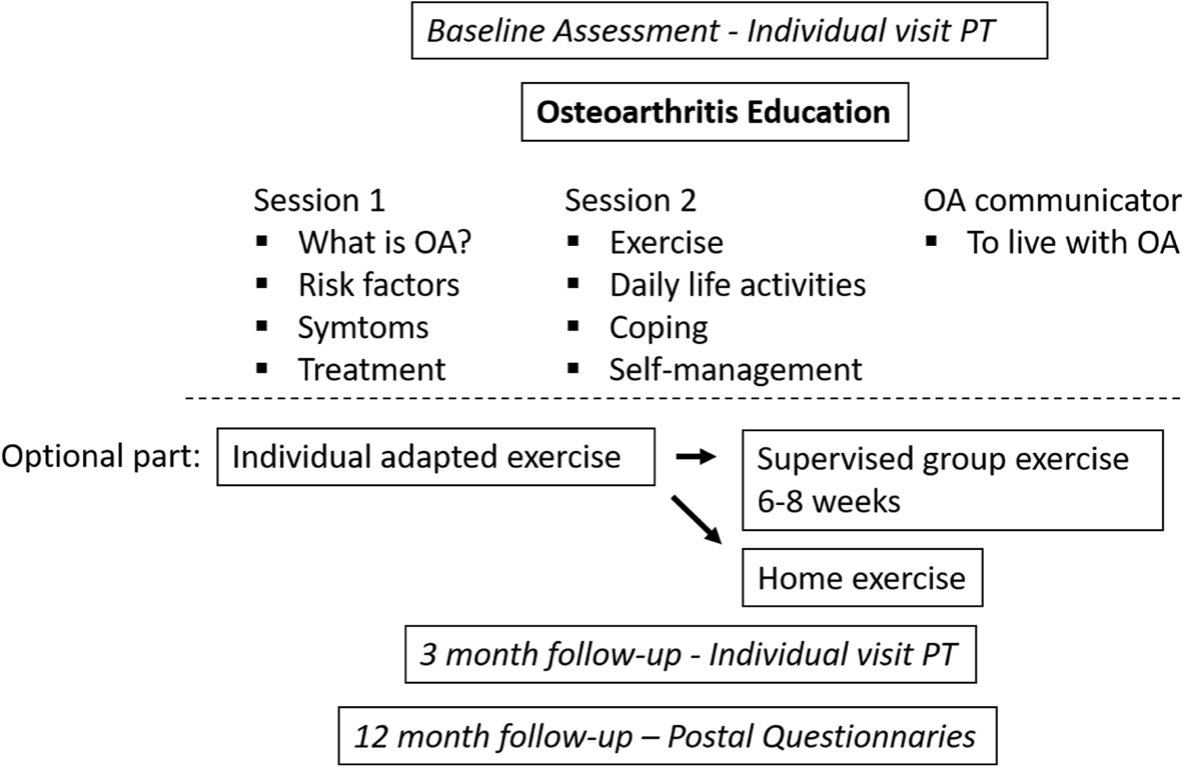
Concept of the education and supervised exercise according to the BOA program

The program combined information delivered by peers and by healthcare professionals with individually adapted exercise (Fig. 1). The program included a minimal intervention of two theoretical group sessions led by a PT. The first session provided information about the pathology and etiology of OA, and treatments according to guidelines. The second session concerned the role of exercise in OA and focused on why exercise is needed, barriers to exercise, how exercise can be incorporated into daily life, and self-management strategies to reduce pain and other symptoms. The third session was not mandatory but was offered by an OA communicator i.e. a patient with OA, who talked about his/her experience of living with OA, as well as his/her personal experience with nonsurgical interventions. The purpose of the theoretical sessions was to explain the mechanisms behind the benefits of the specific exercises and to increase patients’ motivation to exercise and be physical active. After the minimal intervention, patients could choose whether to participate in an individually adapted exercise program or not. The exercise program, based on the patient’s specific needs and goals, was first practiced during a one-on-one session. After that patients could choose to perform the exercises on their own or during PT-supervised exercise classes held twice a week for 6 weeks. Strength training exercises were not specified but were based on the following biomechanical principles: to ensure proximal strength, align the hip–knee–ankle, and achieve good neuromuscular control. The intensity and progress of exercises were based on individual function and capacity, as well as the ability to maintain alignment and control. The model of acceptable pain was used to cope with pain during exercise. If pain occasionally exceeded the acceptable limit, this was used as a learning process, and the dosage of activity was adapted accordingly to achieve an acceptable pain level again. Strategies for incorporating exercise and physical activity into daily living and for maintaining a physically active lifestyle were continuously discussed during the intervention. A home exercise program consisting of one or two daily exercises to be incorporated into daily life and practiced for a few minutes each day was also introduced in parallel to the individual program. To support compliance to an active lifestyle, an individual visit was scheduled 3 months after the first visit, regardless of whether or not the patients chose to participate in an exercise program. The visit after 3 months focused on how exercise and physical activity could be continuously incorporated into daily life. The overall aim of the intervention was to increase patient’s efficacy in self-managing their disease and increasing their level of PA [13]. Physical activity is defined as any bodily movement produced by skeletal muscles that results in energy expenditure, and exercise are defined as a subset of physical activity that is planned, structured, and repetitive and has a final or an intermediate objective of improving or maintaining physical fitness.

### Outcome measures

Physical activity and sedentary time was monitored using a GT1M Actigraph accelerometer (ActiGraph, Pensacola, FL), a small uniaxial accelerometer that measures vertical acceleration and deceleration [24]. The validity and reliability of Actigraph accelerometers have been established in different populations, including OA [25–28]. Participants maintained a daily activity log to record time spent in aquatic and cycling activities, which may not be fully captured by accelerometer. Oral and written instructions were given to the participations to wear the accelerometer on a belt at the natural waistline, on the right hip, aligned with the right axilla, and to wear it continuously (except for aquatic activities) from morning until bedtime for seven consecutive days.

A set of questionnaires were completed, including background variables (sex, age, weight, length, education level, and Charnley category A/B/C), Visual Analog Scale (VAS) for pain, the generic Quality of Life-Instrument (EQ-5D-3 L) and the Arthritis Self Efficacy Scale (ASES).

The Charnley classification is a comorbidity score and categorizes patients into one of three groups: A - one joint with osteoarthritis (unilateral knee or hip); B - bilateral osteoarthritis (both knees or both hips); C - osteoarthritis in multiple joint sites (hip and knee), or presence of any other disease that affects walking ability [29].

The VAS was graded from 0 to 100, where 0 represents “no pain” and 100 represents “worst pain possible.” The VAS is well established in clinical practice and research for measuring pain intensity in OA populations [30].

Health-related quality of life was assessed using the EQ-5D-3 L, which has previously been used for measuring the outcome of intervention in patients with OA [15, 16]. EQ-5D-3 L can be presented as a health profile or as a global health index with a weighted total value (British tariff was used), where the minimum value is − 0.594 and the maximum is 1.0 [31]. This study used only the global health index.

Self-efficacy was assessed by the Arthritis Self-Efficacy Scale (ASES), which is designed to measure confidence in one’s own ability to master and/or reduce a number of implications of chronic arthritis. It consists of 20 statements, divided into three subscales: pain, function, and other symptoms. Each item is scored on a 10-point Likert scale ranging from 10 (very uncertain) to 100 (very certain) [32]. This study used only the subscales for pain and other symptoms. ASES has previously been used to evaluate patient education programs for patients with arthritis [32, 33]. The Swedish version has been proven valid [34].

### Procedure

All patients in the intervention group were assessed by a PT (TJ) at baseline, 2 weeks prior to the first self-management session, and at the 3-month follow-up. Compliance to the intervention is noted by the PT every time the individual had been to theory session or supervised exercise. At the 12-month follow-up, the PT called the participants, and a questionnaire and an accelerometer was sent to the patients by mail. If no response was received within 3 weeks, a reminder was sent. The reference group was assessed by a PT (TJ) at baseline and at the 3-month follow-up. The PT (TJ) conducted a telephone follow-up, and a questionnaire and an accelerometer were sent to the patients.

### Analysis of accelerometer data

The accelerometer was set to collect data using a 10 s epoch (timeframe). After data collection, data was treated according to the following procedures (similar to a previous accelerometer study on a Swedish population [35]); for a day to be considered valid, wear time had to exceed 600 min/day after periods of > 20 min of consecutive epochs with 0 counts had been removed. Only patients with 4 or more days of valid monitoring were included in the subsequent analysis. To calculate the duration of physical activity at different intensities, the commonly used cut-point of < 100 counts per minute [36] was used for sedentary behaviour, and the cut-points developed by Freedson et al. [37] were used for physical activity: 100–1951 counts per minute for light physical activity, 1952–5723 counts per minute for moderate physical activity, 5724 and higher for vigorous physical activity. The sum of all epochs with 1952 counts or more per minute or more was defined as moderate to vigorous physical activity (MVPA).

### Sample size

The sample size calculation was based on a study measuring physical activity in patients with light to moderate grade of OA using an accelerometer [26]. To detect a between group difference of 10 ± 17 min with a probability to make a type I error of 5% and type II error of 20% at least 50 participants in each group was needed. To accommodate for a potential drop out of 20% we aimed to recruit 120 subjects. However, during the course of the study we observed that the actual dropout rate reached 30%. To compensate for the higher-than-expected dropout rate, we included 195 patients in the intervention group.

### Statistics

Statistical analyses were performed using the Statistical Package for the Social Sciences for Windows, version 22 (SPSS, Chicago, IL). Descriptive statistics were presented as mean and standard deviation (SD) or median and interquartile range (IQR). Within each group, change over time (i.e. baseline to 3-month follow-up, and for the intervention group also baseline to 12-month follow-up) was analyzed using the Wilcoxon Signed-Rank test, since data were not normally distributed. Results from this analysis are presented as median and IQR. Concerning the comparison between the intervention group and the reference group, the difference regarding outcome variables between baseline and 3-month follow-up were the dependent variables. Those difference scores were explored for normal distribution using a histogram and judged as approximately normally distributed. Hence, univariate analysis of variance (GLM ANOVA) was used to compare the intervention and reference groups. In the different models, the dependent variables were the computed difference between baseline and 3-month follow-up for: time (minutes) spent in sedentary behavior, low activity, and moderate-to-vigorous physical activity, VAS/pain, EQ-5D, ASES/pain and ASES/other. Fixed factors were: group (intervention/reference), sex, education, joint, Charnley classification. Covariates were age and baseline value for the dependent variable in the model (i e; either time spent in sedentary behavior, low activity, and moderate-to-vigorous physical activity, VAS/pain, EQ-5D, ASES/pain, or ASES/other). The final selection of covariates to be included in the model was performed using a stepwise backward deletion approach, where the least significant variable was removed from the model until only covariates with significant (*p* < 0.2) effects remained in the model [38]. Results are presented as means and mean differences with corresponding 95% confidence intervals. The limit of significance was set to 0.05. The primary outcome was change (from baseline to the 3-month follow-up) in sedentary time, as well as in low and moderate-to-vigorous physical activity time. A dropout analysis was performed, including the variables for sex, age, BMI, Charnley Score, joint (knee/hip), education level, minutes in sedentary behavior, minutes in low activity, minutes in MVP, VAS/pain, EQ-5D, ASES/other or ASES/pain. Chi-squared tests were used for dropout analyses of sex, Charnley classification A/B/C, joint (knee/hip), and education. Mann-Whitney U tests were used for dropout analyses with respect to the following variables: age, physical activity, VAS/pain, EQ-5D and the ASES subscales.

## Results

### Subjects

**Fig. 2:**
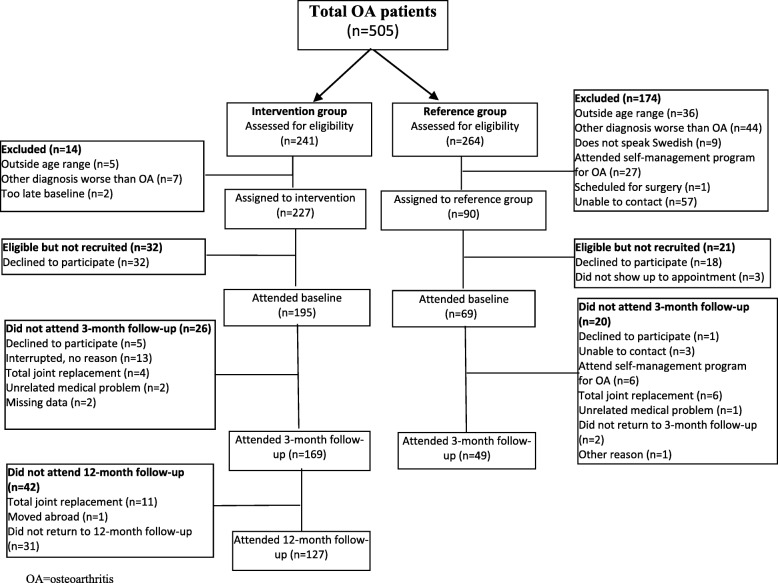
Flowchart of the study

Figure 2 shows a flowchart of the study. Table 1 shows individual characteristics and baseline values for the outcome measures. All patients included in the intervention group attended the minimal intervention, and 98% also received an individual exercise program. Of these latter, 19% participated in supervised exercise 10–12 times, 16% participated 7–9 times, 28% participated 1–6 times, and 37% did not participate in supervised exercise.

**Table 1:** Patient characteristics and baseline data

	Intervention group (*n* = 195)	Reference group (*n* = 69)
Sex, female (%)	64	58
Age, years (mean, SD, range)	60 (10, 29–75)	66 (7, 49–75)
BMI kg/m^2^ (mean SD, range)	28 (5, 18–47)	28 (5, 19–45)
Joint, knee (%)	78	55
Education level; elementary/high school/university (%)	24/40/36	35/36/29
Charnley Category A/B/C (%)	27/41/29	35/33/32
Sedentary, daily minutes (median, IQR)	562 (523–605) *n* = 141	572 (505–599) *n* = 52
Low activity, daily minutes (median, IQR)	180 (150–214) *n* = 141	169 (130–218) *n* = 52
Moderate-vigorous activity, daily minutes (median, IQR)	34 (22–52) *n* = 141	20 (11–30) *n* = 52
VAS/pain (median, IQR)	51 (36–62)	60 (50–70)
EQ-5D index (median, IQR)	0.725 (0.62–0.796)	0.656 (0.159–0.727)
ASES/other symptoms (median, IQR)	68 (53–80)	61 (48–70)
ASES/pain (median, IQR)	60 (46–76)	46 (38–62)

*BMI* body mass index

### Dropout analysis

Participants who dropped out from the intervention group between baseline and the 3-month follow-up, and non-dropout participants (dropout median (IQR) vs. participant median (IQR)) differed along the following variables: age (57 (45–66) vs. 62 (55–66), *p* = 0.042), EQ-5D: (0.73 (0.66–0.80) vs. 0.66 (0.09–0.73), *p* < 0.001) and ASES/other; (55 (42–73) vs. 70 (55–81), *p* = 0.007), Charnley score; (A; 39%, B; 35%, C; 23% vs. A; 26%, B; 43%, C; 31%, *p* = 0.035). Participants who dropped out from the reference group between baseline and 3-month follow-up did not differ from the reference group (data not shown). Participants who dropped out from the intervention group between baseline and 12-month follow-up (dropout median (IQR) vs. participant median (IQR) differed along the following variables: age (59 (48–66) vs. 62 (56–66), *p* = 0.012), VAS/pain (57 (39–69) vs. 50 (31–60), *p* = 0.037), EQ-5D (0.66 (0.29–0.73) vs. 0.73 (0.66–0.80), *p* < 0.001), ASES/other (63 (50–75) vs. 71 (55–83), *p* = 0.08) and joint (hip: 31% vs. 17%; knee: 69% vs. 83%, *p* = 0.045).

### Accelerometer dropout analysis

The number of dropouts from the accelerometer measurement in the intervention group was 54 (28%) after the measurement at baseline. Reasons for dropout were as follows: 45 participants did not have ≥4 valid days of accelerometer wear, and 9 dropouts were due to technical problems with the accelerometer. Participants who dropped out from the intervention group at baseline did not differ from the intervention group (data not shown).

The number of participants who dropped out between baseline and the 3-month follow-up was 59 (30%), all of whom did not have ≥4 valid days of accelerometer wear. These dropouts differed in minutes of low activity (158 (137–207) min vs. 181 (150–216) min, *p* = 0.038). Participants who dropped out from the reference group at baseline (17 participants) and between baseline and the 3-month follow-up (21 participants) did not differ from the rest of the reference group (data not shown). The reason for the dropouts was ≤4 valid days of accelerometer wear.

### Primary outcome (physical activity)

Between baseline and 3-month follow-up, no significant differences were found between the groups with regard to change in minutes spent in sedentary behavior and low or moderate-to-vigorous physical activity (Table 2).

**Table 2:** Differences in mean daily minutes of sedentary behavior, low activity, moderate-vigorous activity, VAS/pain, EQ-5D-index, ASES/pain and ASES/other symptoms for the intervention group compared to the reference group, from baseline to 3-month follow-up, mean difference, 95% confidence interval (CI)

	Intervention group (*n* = 169)	Reference group (*n* = 49)		
	Mean change (CI)	Mean change (CI)	Mean diff (CI)	p
Sedentary (mean minutes)	-2 (-12 to 8) *n* = 112	-11 (-30 to 8) *n* = 28	-9 (-31 to 12)	*0.401*
Low activity (mean minutes)	-8 (-15 to – 2) *n* = 112	-11 (-24 to 2) *n* = 28	-3 (-17 to 12)	*0.707*
Moderate-vigorous activity (mean minutes)	4 (-0.6 to 8) *n* = 112	0.2 (-8 to 9) *n* = 28	-4 (-14 to 6)	*0.460*
VAS/pain (0–100)	-9 (-13 to -6)	4 (-2 to 9)	13 (7 to 19)	< 0.001^*^
EQ-5D	0.03 (-0.004 to 0.07) *n* = 168	-0.14 (-0.19 to -0.08)	-0.17 (-0.24 to -0.10)	< 0.001^*^
ASES/other (10–100)	2 (-0.3 to 5) *n* = 168	-3 (-7 to 1)	-5 (-10 to -0.3)	*0.04* ^*^
ASES/pain (10–100)	5 (2 to 8) *n* = 168	-2 (-7 to 3)	-7 (-13 to -2)	*0.01* ^*^

All measurements are calculated using general linear ANOVA and adjusted for sex, joint (hip/knee), age, education level, Charnley category and baseline values of outcome measures; only potential confounders with significant (*p* < 0.2) effect were kept in each model. *P<0.05

Within-group analysis showed that there was a significant decrease in the number of minutes of low-intensity physical activity in the intervention group when comparing baseline and 3-month follow-up values. This decrease did not persist at the 12-month follow-up, when the change from baseline was no longer significant. However, there was a significant decrease in minutes of moderate-to-vigorous physical activity between baseline and 12-month follow-up, while the decrease at 3 months was not significant. Sedentary time did not change in the intervention group between baseline and the 3 and 12-month follow-ups. In the reference group, no significant change in either activity level was found between baseline and 3-month follow-up. Table 3 shows within-group analyses of change in daily minutes of sedentary, low activity, and moderate-vigorous activity from baseline to 3-month follow-up and 12-month follow-up.

**Table 3:** Median (IQR) of daily minutes of sedentary behavior, low activity, moderate-vigorous activity, from baseline to 3-month follow-up and 12-month follow-up

	Intervention group	P	Reference group	P
	Median (IQR)		Median (IQR)	
Sedentary				
Baseline	562 (523–605) *n* = 141		572 (505–599) *n* = 52	
3 months	556 (507–602) *n* = 129	*0.538*	556 (504–592) *n* = 33	*0.891*
12 months	552 (499–598) *n* = 110	*0.178*		
Low activity				
Baseline	181 (150–214) *n* = 141		169 (130–218) *n* = 52	
3 months	170 (144–205) *n* = 129	*0.023* ^*^	169 (118–220) *n* = 33	*0.072*
12 months	178 (140–228) *n* = 110	*0.633*		
MVP activity				
Baseline	34 (22–52) *n* = 141		20 (11–30) *n* = 52	
3 months	34 (19–52) *n* = 129	*0.998*	21 (10–37) *n* = 33	*0.820*
12 months	32 (18–52) *n* = 110	*0.026* ^*^		

MVP = moderate-vigorous physical activity. Wilcoxon Signed-Rank test were used, and *p*-values were calculated from baseline to three-month follow-up and baseline to 12-month follow-up

^*^P < 0.05

No changes between baseline and 3-month follow-up were found in the daily activity log with regards to minutes spent in biking, water activity and gym exercise.

### Secondary outcomes (VAS/pain, EQ-5D, ASES/pain, ASES/other)

When comparing the intervention and the reference group with regards to changes in the secondary outcomes (i.e. VAS/pain, EQ-5D, ASES/other and ASES/pain), we found that as a whole, individuals in the intervention group showed significantly greater change for all outcomes (mean diff; 95% CI; significance): VAS/pain (13; 7 to 19; *p < 0.001*), EQ-5D quality of life (− 0.17; − 0.24 to − 0.10; *p < 0.001*), ASES/other (− 5; − 10 to − 0.3; *p < 0.04*), and ASES/pain (− 7; − 13 to − 2; *p < 0.01*) (Table 2).

Within the intervention group we found significant improvements for VAS/pain, EQ-5D, ASES/other and ASES/pain. In the intervention group, the improvement in VAS/pain and EQ-5D persisted at the 12-month follow-up (Table 4).

**Table 4:** Median (IQR) of VAS/pain, EQ-5D-index, ASES/pain and ASES/other symptoms, from baseline to three-month follow-up and 12-month follow-up

	Intervention group	P	Reference group	P
	Median (IQR)		Median (IQR)	
VAS/pain				
Baseline	51 (36–62) *n* = 195		60 (50–70) *n* = 69	
3 months	37 (21–54) *n* = 169	*< 0.001* ^*^	62 (37–71) *n* = 49	*0.668*
12 months	38 (21–57) *n* = 127	*< 0.001* ^*^		
EQ-5D				
Baseline	0.73 (0.62–0.80) *n* = 193		0.66 (0.16–0.73) *n* = 69	
3 months	0.73 (0.69–0.80) *n* = 169	*< 0.001* ^*^	0.52 (0.09–0.73) *n* = 49	*0.177*
12 months	0.73 (0.69–0.80) *n* = 127	*0.009* ^*^		
ASES/other				
Baseline	68 (53–80) *n* = 193		61 (48–70) *n* = 69	
3 months	76 (61–86) *n* = 169	*< 0.001* ^*^	56 (46–81) *n* = 47	*0.937*
12 months	71 (55–83) *n* = 125	*0.409*		
ASES/pain				
Baseline	60 (46–76) *n* = 193		46 (38–62) *n* = 69	
3 months	74 (56–86) *n* = 169	*< 0.001* ^*^	50 (32–70) *n* = 47	*0.391*
12 months	68 (46–84) *n* = 123	*0.109*		

MVP = moderate-vigorous physical activity. Wilcoxon Signed-Rank test were used, and *p*-value were calculated from baseline to three-month follow-up and baseline to 12-month follow-up

^*^P < 0.05

In the reference group, no significant changes were found regarding VAS/pain, EQ-5D, ASES/other or ASES/pain between baseline and 3-month follow-up **(**Table 4**).**

## Discussion

To the best of our knowledge, this is the first study that has evaluated physical activity level measured with an accelerometer before and after a self-management program with education and supervised exercise for individuals with knee or hip OA. Our data suggest that participating in education and supervised exercise following the BOA program significantly reduced pain, increased quality of life, and increased self-efficacy—however, it did so without a concomitant change of physical activity level.

Improvements in pain and quality of life persisted after 12 months, indicating that education and supervised exercise following the BOA program had sustained long-term effects for patients with knee or hip OA.

Most people with OA have fluctuating symptoms and an improvement might not only affect pain relief but also how patients cope with pain in their daily life and are able to continue to be physically active. Factors such as sex, age, BMI, and disease-related factors such as pain and low self-efficacy are associated with lower levels of physical activity [20, 39]. Although participation in the intervention arm of this study achieved pain relief and increased self-efficacy and quality of life, all of which are in line with previous findings [14–18, 40], it did not decrease sedentary time or increase physical activity time. Webber et al. found similar results for OA patients who underwent total joint replacement; these patients did not change their activity level after surgery, despite achieving pain relief [41]. Interestingly, the median value for physical activity (at the moderate-to-vigorous level) was 34 min/day at baseline, which is 3 min higher than for the general population of Sweden [35]. The supervised exercise in our self-management program was individually tailored, with a focus on strength training. The fact that the accelerometer underestimates strength training may mean that the patients actually increased their physical activity level in ways that were not detected.

As has been shown previously, changing health behavior and maintaining a healthy lifestyle requires a continuous commitment [42]. Individuals with chronic diseases such as OA often have to make permanent lifestyle changes and create new behavior patterns, and the goal in physical therapy is often to make the individuals as independent as possible regarding physical activity without help from a PT. Physical interventions to increase physical activity should include the following behavioral approaches: setting goals for physical activity and self-monitoring of progress, building social support for new behavior patterns, reinforcing such behavior through self-reward, enabling structured problem-solving aiming to maintain the behavior change, and preventing relapse into sedentary behavior [43]. All these parameters are included in the intervention but it may need additional focus on increasing the level of physical activity.

In this study, education and supervised exercise for patients with knee and hip OA was designed according to existing evidence-based guidelines [44]. While it is possible to exercise at home, participants were encouraged to participate in the supervised exercises, since a meta-analysis showed that 12 or more supervised sessions are twice as effective as fewer than 12 supervised sessions [45]. Only 19% of the study population participated in 10–12 supervised exercise sessions. Thus, greater compliance with supervised exercise might have increased the difference between the intervention group and the reference group.

The most common reason for dropping out from the intervention group was failure to complete the self-management program. Compared to the intervention group, the dropouts were younger and healthier, but rated their quality of life and self-efficacy as lower. These differences may affect our results negatively, since younger individuals seems to benefit more from OA intervention than older individuals (unpublished data from the BOA registry). The fact that education and supervised exercise following the BOA program did not attract younger persons has previously been described [46]; one reason may be that younger persons are working and thus finding it harder to take time out of their schedule to participate in education and supervised exercise. With the development of an OA program based on the BOA guidelines but delivered online [47], many of these barriers to “in-person” treatment may be overcome.

Our study includes some limitations that need to be addressed. First, participants were not randomized into the treatment group vs. non-treatment reference group. The two groups were not matched in terms of sex, age, and location of OA (knee or hip), and we did observe a difference between the two groups with respect to baseline values. We adjusted for these discrepancies using a statistical model (GLM-ANOVA). Despite these limitations, we observed a within-group change for the intervention group but not for the reference group, which supports our conclusion. Importantly, both the intervention group and the reference group were recruited and assessed in clinical practice, increasing the generalizability of results. Second, the physiotherapist was not blinded to the two groups, and the intervention group saw a physiotherapist between 5 and 18 times versus the reference group that only saw a physiotherapist once. More frequent PT-led training may improve outcomes [48] and could thus be a reason for the between-group differences seen in our study. Our self-reported results may be affected by patients wanting to “do well” and to please healthcare providers by reporting a false or exaggerated improvement. However, despite having no PT involvement between 3 and 12 months, the result persisted at the 12-month follow-up.

Finally, education and supervised exercise following the BOA program seems to be a safe treatment and no complications were reported. Future work in OA self-management needs to focus on patient engagement to improve physical activity, in addition to improvements in pain and function.

## Conclusion

Participation in an education and exercise program according to the Swedish BOA program neither decreased the average amount of sedentary time nor increased their level of physical activity. However, participation in such a program did result in decreased pain, increased quality of life, and increased self-efficacy.

## Abbreviations

ASES: Arthritis Self-Efficacy Scale
BMI: Body Mass Index
BOA: Better management of patients with osteoarthritis
IQR: Interquartile range
OA: Osteoarthritis
PT: Physiotherapist
SD: Standard deviation
TJ: Therese Jönsson
VAS: Visual Analog Scale
